# Case Report: A rare *RUNX1* rearrangement resulting from t(8;21)(p12;q22) in acute myeloid leukemia with plasmacytoid dendritic cell expansion

**DOI:** 10.3389/fonc.2025.1672416

**Published:** 2025-10-01

**Authors:** Jinying Gong, Jialong Liu, Lisha Lu, Jiru Wei, Xue Wu, Junyan Zou, Yuan Feng, Guoqing Zhu, Jing Han

**Affiliations:** ^1^ State Key Laboratory of Experimental Hematology, National Clinical Research Center for Blood Diseases, Haihe Laboratory of Cell Ecosystem, Institute of Hematology & Blood Diseases Hospital, Chinese Academy of Medical Sciences & Peking Union Medical College, Tianjin, China; ^2^ Hematology Pathology Diagnostic Center, Tianjin Union Precision Medical Diagnostics Co., Ltd, Tianjin, China; ^3^ Department of Medical Oncology, Beidahuang Industry Group General Hospital, Harbin, China

**Keywords:** RUNX1 rearrangement, plasmacytoid dendritic cell expansion, RUNX1A, acute myeloid leukemia, RUNX1 mutation

## Abstract

In recent years, acute myeloid leukemia with plasmacytoid dendritic cell expansion (pDC-AML) has been recognized as a rare provisional subtype of AML, comprising approximately 3–5% of all reported cases and associated with a poorer clinical outcome compared with non–pDC-AML. Both *RUNX1* mutations and rare rearrangements can lead to either complete loss or dominant-negative inhibition of RUNX1 function in pDC-AML, which may play a pivotal role in the aberrant expansion or malignant transformation of plasmacytoid dendritic cells (pDCs). To date, only two cases of pDC-AML with rare *RUNX1* rearrangements have been reported. Herein, we reported a rare *RUNX1* rearrangement resulting from t(8;21)(p12;q22) in a patient with pDC-AML, leading to the truncated RUNX1 that exhibit structural and functional similarities to RUNX1A and may act as a dominant-inhibitor of wild-type RUNX1. Given the poor prognosis associated with this subtype, CD123-targeted therapy, such as tagraxofusp-erzs, alone or in combination with agents like azacitidine and venetoclax, may represent a rational therapeutic approach. To our knowledge, this represents the third case report of *RUNX1* rearrangement in pDC-AML and may provide valuable insights for future research.

## Introduction

In recent years, acute myeloid leukemia with plasmacytoid dendritic cell expansion (pDC-AML) has been recognized as a rare provisional subtype of AML, comprising approximately 3–5% of all reported AML cases and associated with poor clinical outcomes (1, 2). Mutations in the Runt-related transcription factor 1 (*RUNX1*) gene are the most frequently observed genetic alterations in pDC-AML, occurring in approximately 70% of cases, and are potentially associated with the malignant transcriptional program of plasmacytoid dendritic cells (pDCs) (1–3). Notably, *RUNX1* rearrangements in pDC-AML are relatively rare, with only two cases reported to date (1, 4).

RUNX1, also known as AML1, CBFA2, or PEBP2aB, is a key transcription factor essential for the emergence of definitive hematopoiesis and the precise regulation of adult hematopoiesis, whose dysregulation can lead to aberrant hematopoietic function (5). Due to the involvement of two distinct promoters and alternative splicing in RUNX1 synthesis, the protein exists in three major isoforms: RUNX1A, RUNX1B, and RUNX1C. However, the functional relationships among these isoforms have not been fully elucidated and remain unknown (6). Recurrent genetic alterations in *RUNX1* gene, primarily including monoallelic rearrangement as well as monoallelic or biallelic mutations, have been identified in both hereditary and sporadic hematologic disorders, particularly in AML or myelodysplastic syndromes (MDS) (5, 7).

*RUNX1* rearrangements can give rise to two types of fusion events: gene-gene fusions, which may be either in-frame or out-of-frame, and gene-intergenic fusions (8). In-frame fusions typically generate novel fusion proteins that may act as oncogenic drivers, whereas out-of-frame and gene-intergenic fusions are more likely to result in truncated upstream gene products or potential haploinsufficiency of both genes involved (9, 10).

To date, more than 40 recurrent chromosomal rearrangements involving *RUNX1* gene have been identified in AML/MDS. Among these, the most frequent partner genes are *RUNX1T1* (8q21) and *MECOM* (3q26, including *MDS1* and *EVI1*) (6). The *RUNX1*::*RUNX1T1* fusion is associated with favorable prognosis, whereas the *RUNX1*::*MECOM* fusion and other rare *RUNX1* fusion may be associated with unfavorable prognosis (4). Since the concept of pDC-AML was proposed around 2018 and was not recorded in the WHO classification until 2022, earlier reports of *RUNX1* rearrangements may have lacked the information regarding pDCs (1, 11). This retrospective gap likely limits our current understanding of the association between *RUNX1* rearrangements and pDC-AML.

Herein, we report a rare *RUNX1* rearrangement resulting from t(8;21)(p12;q22) in a patient with pDC-AML, leading to the truncated RUNX1 that exhibit structural and functional similarities to RUNX1A. The truncated RUNX1 may act as a dominant inhibitor of wild-type RUNX1, potentially playing a pivotal role in the aberrant expansion or malignant transformation of pDCs. To our knowledge, this represents the third case report of *RUNX1* rearrangement in pDC-AML and may provide valuable insights for future research.

## Case presentation

**Figure 1:**
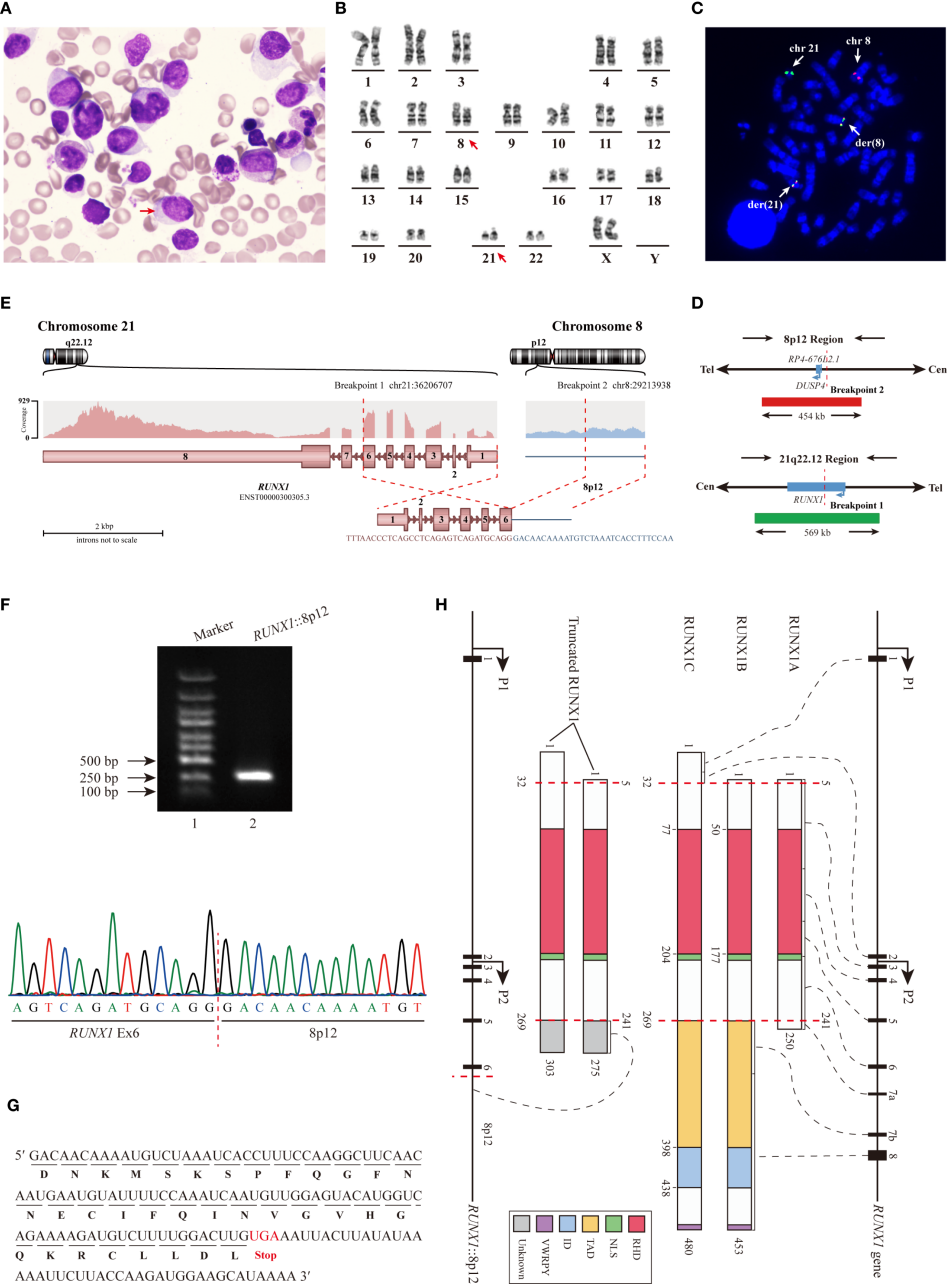
Identification of the novel *RUNX1* rearrangement. **(A)** BM biopsy revealed marked proliferation of promonocytes and immature monocytes. **(B)** Conventional chromosome analysis demonstrated the t(8;21)(p12;q22) involving the *RUNX1* gene. **(C)** Detection of *RUNX1* rearrangement by metaphase FISH Using LSI *RUNX1*/*DUSP4* Dual Color Dual Fusion Probes. **(D)** Schematic diagram of LSI *RUNX1*/*DUSP4* Dual Color Dual Fusion Probes. **(E)** RNA-seq identified the *RUNX1*::8p12 fusion. **(F)** RT−qPCR analysis (Lane 2) and subsequent Sanger sequencing confirmed the *RUNX1*::8p12 fusion. **(G)** Analysis of the mRNA sequence revealed an additional segment (34 aa) in the truncated *RUNX1* transcript. **(H)** Schematic diagram illustrated the three primary RUNX1 isoforms, and the truncated RUNX1 isoforms identified in this study. Abbreviation: NLS, nuclear localization signal.

A 51-year-old woman presented to the hospital with a six-month history of weakness and a two-week history of gingival swelling and pain. Her complete blood count indicated the following: red blood cell count of 2.96×10^12^/L, hemoglobin of 96 g/L, white blood cell count of 10.49×10^9^/L, with 38.3% monocytes and 27.9% lymphocytes, and platelet count of 181×10^9^/L. Bone marrow (BM) biopsy revealed marked proliferative activity, with a decreased proportion of granulocytes and lymphocytes, and a relative increase in monocytes. Notably, 43% of the monocytes were identified as promonocytes and immature forms, and Auer rods (red arrow) were observed in some of these cells (**Figure 1A**). Physical examination and computed tomography (CT) scan revealed no cutaneous lesions or lymphadenopathy, and no hepatosplenomegaly was observed.

Flow cytometry analysis revealed 7.85% of abnormal myeloid blasts (CD117+, CD34+, CD13+, CD33+, CD123+, HLA-DR^dim^+ and CD38^dim^+) and 40.69% of abnormal immature monocytes (CD33^bri^+, HLA-DR^bri^+, CD38+, CD13+, CD123+, CD36+, partially CD64+, partially CD11b+, partially CD15+, partially CD14+ and CD4^dim^+). Additionally, 5.70% of pDCs were detected, with the following phenotype: CD123^bri^+, HLA-DR^bri^+, CD303+, CD304+, TDT-, CD34-, CD56-, and CD4+. The Wilm tumor gene-1 (*WT1*) expression was positive, with a quantitative value of 17.82%.

Conventional chromosome analysis revealed an abnormal karyotype described as 46,XX,t(8;21)(p12;q22)[20] (**Figure 1B**). The metaphase fluorescence *in situ* hybridization (FISH) analysis utilizing LSI *RUNX1*/*RUNX1T1* Dual Color Dual Fusion Probes confirmed the presence of *RUNX1* rearrangement. Part of the *RUNX1* signal on chromosome 21q22 was translocated to the derivative chromosome 8. Initially, we proposed that *DUSP4* gene might be the potential partner of *RUNX1* gene, and the metaphase FISH utilizing LSI *RUNX1*/*DUSP4* Dual Color Dual Fusion Probes showed a 78% positive signal (**Figures 1C, D**). However, RNA sequencing (RNA-seq) analysis revealed certain biases, indicating that *DUSP4* was not the partner gene.

RNA-seq analysis identified the *RUNX1* rearrangement event (**Figure 1E**). Exon 6 (Ex6) of the *RUNX1* gene was fused to an intergenic region on chromosome 8p12, located approximately 5,766 base pairs upstream of the *DUSP4* gene. RT−qPCR analysis and subsequent Sanger sequencing confirm the fusion between *RUNX1* and the intergenic region (**Figure 1F**), resulting in the truncated RUNX1 fused with an additional 34 amino acid (aa) peptide of unknown function (**Figure 1G**). The reciprocal fusion transcript was not detected. Quantitative analysis revealed that the expression level of the fusion transcript was 32.62%.

Unfortunately, the patient was transferred to another hospital and passed away two months later, limiting the availability of further treatment information.

## Discussion

In the fifth edition of the World Health Organization classification of hematolymphoid tumors (WHO-HEM5), neoplasms involving pDCs are classified into two entities: blastic plasmacytoid dendritic cell neoplasm (BPDCN) and mature plasmacytoid dendritic cell proliferation (MPDCP) associated with myeloid neoplasms (11). However, given the typical association of MPDCP with other myeloid neoplasms, the International Consensus Classification (ICC) has not formally recognized MPDCP as a distinct myeloid entity (12). Furthermore, the definition of MPDCP remains ambiguous, and the use of the term “mature” is considered inappropriate, as MPDCP cases associated with AML often comprise pDCs at early to intermediate stages of differentiation (3). The introduction of the term pDC-AML aimed to distinguish cases of MPDCP associated with AML from those involving chronic myelomonocytic leukemia (CMML) or MDS.

pDC-AML is typically characterized by cross-lineage antigen expression, adverse risk stratification, and poor outcomes, with a high frequency of *RUNX1* mutations and upregulation of pDC transcriptional programs expression (1, 13). The pDCs display a spectrum of maturation from early pDCs to fully mature pDCs and are thought to originate from early pDC progenitors (14). Notably, the pDCs are predominantly at an early maturation stage. In two related studies, the expression of CD34 in pDCs from patients with pDC-AML was reported at 61% (25/41) and 98% (52/53), respectively (1, 2). In our case, pDCs were negative for CD34, suggesting that they may represent intermediate to late stages of maturation. At present, it remains unclear whether pDCs at different maturation stages have differential effects in pDC-AML.

In the study by Wenbin Xiao et al. (1), 78% (32/41) of the pDC-AML cases exhibited alterations in the *RUNX1* gene, including 29 patients with *RUNX1* mutations, 2 with atypical *RUNX1* rearrangements, and 1 with a deletion in the region including *RUNX1*. Combined with data from two additional studies, the frequency of *RUNX1* mutations in pDC-AML is approximately 70%, markedly higher than the 6~15% reported in overall AML (2, 3, 15) Based on the available data, the frequency of rare *RUNX1* rearrangements in pDC-AML is approximately 5%, compared to less than 1% in overall AML, showing a significant increase comparable to that of *RUNX1* mutations (1, 4). Therefore, we propose that rare *RUNX1* rearrangements may also be associated with the development of pDC-AML.

As shown in **Figure 1H**, all three RUNX1 isoforms share a conserved 128 aa runt homology domain (RHD), which mediates heterodimerization with core-binding factor subunit beta (CBFB) and facilitates DNA binding to form a transcription factor complex. Additionally, RUNX1B and RUNX1C also contain a transactivation domain (TAD), an inhibitory domain (ID) and the conserved C-terminal pentapeptide motif, VWRPY. The TAD and ID regulate gene activation and repression by interacting with various proteins, while the VWRPY motif mediates transcriptional repression through interactions with Groucho/TLE transcriptional corepressors (5). RUNX1B and RUNX1C exhibit similar functions, whereas RUNX1A exerts a dominant-negative effect on both isoforms (16). Alterations in RUNX1 can generally be classified into two categories (1): those that disrupt the RHD, leading to complete loss of RUNX1 function, and (2) those that retain an intact RHD but disrupt the TAD, ID, or VWRPY motif, conferring dominant negative activity to wild-type RUNX1 (7).

Rare *RUNX1* rearrangement resulting from t (8,21)(p12;q22) in our case led to the RUNX1 truncation. The predicted proteins retain the RHD but lacks the TAD, ID and VWRPY, thereby exhibiting structural and functional similarities to RUNX1A that may act as a dominant-inhibitor of wild-type RUNX1 by competing for DNA binding and interaction with CBFB (4, 16). In pDC-AML, both *RUNX1* mutations (**Supplementary Table 1**) and rare rearrangements (**Table 1**) can lead to either complete loss or dominant-negative inhibition of RUNX1 function, which may play a pivotal role in the aberrant expansion or malignant transformation of pDCs. Additionally, functional suppression of RUNX1 may also occur in pDC-AML cases without detectable *RUNX1* alterations.

As shown in **Table 1**, rare *RUNX1* rearrangements, including both gene-gene and gene-intergenic fusions, are likely associated with poor prognosis. Notably, approximately half of the gene–gene fusions are out-of-frame. Since the concept of pDC-AML was proposed around 2018 and was not recorded in the WHO classification until 2022, earlier case reports may lack the information regarding pDCs (1, 11). To date, only two cases of pDC-AML with *RUNX1* rearrangement have been reported (4). Compared with non–pDC-AML, pDC-AML is associated with a poorer prognosis. At present, pDC-AML remains a provisional entity within the broader category of AML, and consensus on its treatment is yet to be reached, necessitating further research.

**Table 1:** The clinical outcomes in AML/MDS patients with rare RUNX1 rearrangements.

Patient	Age/sex	Partner gene	Frameshift	Reciprocal fusion	Follow-up	Survival status	Doi
1	NA	(Ex8) *RUNX1*::*EAP* (*RPL22P1*, Ex1)	Out-of-frame	NA	NA	NA	10.1073/pnas.90.16.7784
2	76/M	(Ex6) *RUNX1*::*CPNE8* (Ex3)	Out-of-frame	No	NA	D	10.1038/sj.leu.2403048
3	74/M	(Ex6) *RUNX1*::*PRDX4* (Ex2)	In-frame	No	5 m	D	10.1002/gcc.20050
4	77/F	(Ex6) *RUNX1*::*PRDM16* (Ex1)	Both	Yes	2 m	R	10.1002/gcc.20241
5	78/M	(Ex6) *RUNX1*::*FOG2* (*ZFPM2*, Ex6)	In-frame	Yes	NA	D	10.1182/blood-2004-07-2762
6	7/M	(Ex6) *RUNX1*::*USP42* (Ex2)	In-frame	No	10 yr	A	10.1038/sj.leu.2404076
7	56/M	(Ex5/6) *RUNX1*::*TRPS1* (Ex1)	In-frame	No	2 yr	D	10.1182/blood-2006-01-031781
8	73/F	(Ex5) *RUNX1*::*CBFA2T3*(*MTG16*, Ex4)	In-frame	NA	NA	NA	10.1016/j.cancergencyto.2008.04.011
9	NA	(Ex5/6) *RUNX1*::*LPXN* (Ex8/9)	Both	Yes	NA	NA	10.1002/gcc.20704
10	1/M	(Ex3) *RUNX1*::*ZNF687* (5`UTR)	In-frame	No	NA	NA	10.1002/gcc.20355
11	68/M	(Ex5) *RUNX1*::*YTHDF2* (Ex6)	In-frame	No	NA	NA	
12	81/M	(Ex5/6) *RUNX1*::*SH3D19* (Ex2)	In-frame	No	NA	NA	
13	63/F	(Ex5/6) *RUNX1*::LRP16 (MACROD1) (Ex2)	In-frame	Yes	2 yr	R	10.1111/j.1600-0609.2007.00858.x
14	69/M	(Ex6/7) *RUNX1*::*CBFA2T2* (Ex2/3)	Out-of-frame	No	7 m	D	10.1038/leu.2010.106
15	62/M	(*NOL4L*, Ex5/6) *C20orf112*::*RUNX1* (Ex3/4/5)	Out-of-frame	Yes	8 m	D	
16	63/M	(Ex6) *RUNX1*::*CLCA2* (Ex2/5/6)	Out-of-frame	No	1 m	D	10.1016/j.cancergencyto.2010.07.116
17	82/F	(Ex6) *RUNX1*::reversed *UPK3B*::*DTX2* (Ex10)	NA	NA	NA	NA	10.1007/s12185-012-1112-z
18	78/M	(Ex5/6) *RUNX1*::*KIAA1549L*(*C11orf41*, Ex13)	In-frame	No	2 yr	D	10.1016/j.cancergen.2012.10.001
19	43/F	(Ex1) *RUNX1*::*SV2B* (Ex2)	Out-of-frame	No	NA	NA	10.1002/gcc.22105
20	54/F	(Ex25/26) *CHD1*::*RUNX1 (Ex6)*	Out-of-frame	No	2 yr	D	10.1186/s12943-015-0353-x
21	76/M	(Ex5) *RUNX1*::*TMEM48*(*NDC1*, Ex3)	Out-of-frame	No	1 yr	D	10.1038/onc.2015.70
22	69/M	(Ex7) *RUNX1*::SYNE2 (Ex62)	Out-of-frame	Yes	>2 yr	A	10.1016/j.cancergen.2017.07.002
23	50/M	(Ex5/6) *RUNX1*::*TACC1* (Ex6)	Out-of-frame	No	~1 m	D	10.1111/bjh.16444
24	74/F	(Ex7) *RUNX1*::*ZFPM2* (Ex6)	In-frame	No	14 m	D	10.1002/gcc.22901
25	80/M	(Ex2) *RUNX1*::*RCAN1* (Ex2)	Out-of-frame	Yes	5 m	D	
26	66/M	(Ex8) *RUNX1*::*DSCAM* (Ex4)	In-frame	Yes	2 m	D	
27	23/F	(Ex8) *RUNX1*::*LINC00478* (Ex5)	Out-of-frame	No	NA	NA	
28*	61/F	(Ex2) *RUNX1*::*PLAG1* (Ex2)	Out-of-frame	Yes	NA	NA	
29	57/M	(Ex2) *RUNX1*::*WSB2* (Ex2)	In-frame	No	19 m	D	
30	49/M	(Ex6) *RUNX1*::*KIF2C* (Ex7)	In-frame	Yes	NA	NA	
31	33/F	(Ex2) *RUNX1*::*CRYZL1* (Ex2)	In-frame	No	7 m	D	
32	69/F	(Ex7) *RUNX1*::*SEPT7* (*SEPTIN7*, Ex2)	In-frame	No	NA	NA	
33*	75/F	(*AFG2B*, Ex2) *SPATA5L1*::*RUNX1* (Ex2)	NA	No	8 m	D	
34	1/M	(Ex5) *RUNX1*::*ZNF423* (Ex4)	NA	No	NA	NA	10.1038/s41375-023-02024-6
35	79/F	(Ex2) *RUNX1*::*WIF1* (Ex3)	Out-of-frame	No	1 yr	D	10.1007/s12308-024-00597-4
36	32/M	(Ex6) *RUNX1*::*STX2* (Ex5)	In-frame	No	NA	NA	10.1002/mc.23850
37	71/M	(Ex6) *RUNX1*::*MIR99AHG* (Ex2/4)	NA	No	~1 m	D	10.1002/gcc.23272
38	NA	(Ex5/6) *RUNX1*::*AMP19* (intergenic sequence)	Out-of-frame	NA	NA	NA	10.1182/blood.V97.7.2168
39	23/F	*RUNX1*::10q24 (intergenic sequence)	NA	NA	1 yr	D	10.1007/s12032-011-9890-3
40	7/F	(Ex7) *RUNX1*::6q25 (intergenic sequence)	Out-of-frame	No	6 m	D	10.3892/or.2016.5119
41	2/M	(Ex1) *RUNX1*::5q13 (intergenic sequence)	NA	Yes	9 m	R	10.1186/s12943-018-0881-2
42*	51/M	(Ex6) *RUNX1*::8p12 (intergenic sequence)	In-frame	No	~2 m	D	this case

Ex, exon; NA, not available; D, deceased; R, relapsed; A, alive; *: pDC-AML.

Similar to BPDCN, pDC-AML is characterized by pDC expansion, which is associated with poor prognosis. However, the pattern of organ involvement differs between the two entities: BPDCN most commonly presents with cutaneous lesions, whereas pDC-AML predominantly affects the BM and only rarely involves the skin (1, 17). Consequently, therapeutic strategies aimed at eliminating pDCs are considered critical for both BPDCN and pDC-AML, and interleukin-3 receptor α chain (IL3RA or CD123)-targeted therapy represents a promising approach. CD123 is aberrantly overexpressed across a broad spectrum of hematologic malignancies, especially in BPDCN and AML (18). In AML, CD123 can be found in blasts, CD34+ progenitors, CD34+CD38- leukemia stem cells (LSCs), whereas normal HSCs have little (less than 1%) to no CD123 expression (18). This highly restricted expression profile makes CD123 an ideal target both for diagnostic applications and therapeutic interventions in AML, particularly in the context of pDC-AML.

Currently, tagraxofusp-erzs is the only CD123-targeted agent approved by the US Food and Drug Administration (FDA) and has demonstrated robust clinical efficacy in BPDCN, while several other CD123-targeting agents are in development or undergoing evaluation in clinical trials (18, 19). Preclinical studies in murine models have shown that tagraxofusp-erzs can effectively eliminate pDCs in pDC-AML and reduce leukemic burden, mirroring its activity in BPDCN (1). These findings suggest that CD123-directed therapy, alone or in combination with other anti-leukemic agents, may improve outcomes in pDC-AML, although its clinical efficacy has yet to be validated. Encouragingly, ongoing phase I/II clinical trials are evaluating combinations of tagraxofusp-erzs with azacitidine and venetoclax in untreated, relapsed, or refractory AML including cases of pDC-AML (NCT03113643), which may provide further insight into the therapeutic potential of CD123-targeted strategies in this disease (20). In the absence of patient transfer, we would favor this therapeutic strategy for the current case. Moreover, the t (8,21)(p12;q22) or *RUNX1* rearrangement may serve as a potential marker for measurable residual disease (MRD) monitoring and assessment of therapeutic response.

In conclusion, current research on pDC-AML remains limited, and the molecular mechanisms underlying the development of pDCs are still poorly understood. Based on previous studies, signaling pathways involving the *RUNX1* gene may play a critical role. Both *RUNX1* mutations and rare rearrangements can result in either complete loss of RUNX1 function or exert dominant-negative effects on the wild-type RUNX1. However, the precise impact of these alterations on pDC differentiation and expansion remains unclear. It is also unknown whether certain mutations in other genes might promote pDC expansion indirectly by suppressing wild-type RUNX1. Given the poor prognosis associated with pDC-AML, further elucidation of its molecular pathogenesis is essential to guide the development of targeted therapeutic strategies and improve clinical outcomes.

## Data Availability

The original contributions presented in the study are included in the article/**Supplementary Material**. Further inquiries can be directed to the corresponding authors.
